# Supplementary material for: Relationship between emotional intelligence and empathy towards humans and animals
Source: PeerJ. 2021 Apr 16;9:e11274. doi: 10.7717/peerj.11274 (PMC8054732; doi:10.7717/peerj.11274)
Supplement: Supplemental Information 2 [file peerj-09-11274-s002.docx]

**Codebook**

Gender = 1 means male, Gender = 2 means female.

Pets_adulthood= 1 means Pet owner sample, Pets_adulthood= 2 means Non-pet owner sample
